# Development and Evaluation of a Nested PCR for Improved Diagnosis and Genetic Analysis of Peste des Petits Ruminants Virus (PPRV) for Future Use in Nascent PPR Eradication Programme

**DOI:** 10.3390/ani11113170

**Published:** 2021-11-05

**Authors:** Mana Mahapatra, Martin Mayora Neto, Asha Khunti, Felix Njeumi, Satya Parida

**Affiliations:** 1 The Pirbright Institute, Ash Road, Pirbright, Woking GU24 0NF, UK; mana.mahapatra@pirbright.ac.uk (M.M.); martinneto@hotmail.com (M.M.N.); Ashakhunti@hotmail.co.uk (A.K.); 2 Food and Agriculture Organization of the United Nations (FAO), Viale delle Terme di Caracalla, 00153 Rome, Italy; felix.njeumi@fao.org

**Keywords:** peste des petits ruminants virus, PPR, RT-PCR, rapid detection, diagnostic, genetic analysis, eradication program

## Abstract

**Simple Summary:**

Peste des petits ruminants (PPR) is a highly contagious and economically important viral disease of small ruminants and a large number of wildlife species. The causative agent, PPR virus (PPRV) is mainly circulating across large areas in Africa, the Middle East and Asia and is, fortunately, currently subject to a global eradication program. A robust vaccination program along with a rapid and accurate laboratory diagnosis play a major role in the effective control and eradication of the disease. Furthermore, a genetic analysis of the circulating PPRV can help to identify the epidemiological linkage of the virus spread between neighboring countries. Here, we have developed an improved polymerase chain reaction (PCR) that can detect low viral loads and help in genetic identification, particularly in resource-limited settings.

**Abstract:**

Peste des petits ruminants (PPR) is a highly contagious viral disease of small ruminants caused by PPR virus (PPRV). PPR is endemic in Asia, the Middle East and across large areas of Africa and is currently targeted for global eradication by 2030. The virus exists as four different lineages that are usually limited to specific geographical areas. However, recent reports of spread of PPRV, in particular of lineage IV viruses to infection-free countries and previously PPR endemic areas are noteworthy. A rapid and accurate laboratory diagnosis and reports on its epidemiological linkage for virus spread play a major role in the effective control and eradication of the disease. Currently, molecular assays, including conventional reverse transcription-polymerase chain reaction (RT-PCR) and real-time RT-PCR (RT-qPCR) are usually used for diagnosis of PPR while the sequencing of part of the nucleocapsid gene is usually carried out for the viral lineage identification. However, it is difficult to diagnose and sequence the genetic material if the animal excreted a low level of virus at the initial stage of infection or if the PPRV is degraded during the long-distance transportation of samples to the reference laboratories. This study describes the development of a novel nested RT-PCR assay for the detection of the PPRV nucleic acid by targeting the N-protein gene, compares the performance of the assay with the existing conventional RT-PCR and also provides good-quality DNA suitable for sequencing in order to identify circulating lineages. The assay was evaluated using cell culture propagated PPRVs, field samples from clinically infected animals and samples from experimentally infected animals encompassing all four lineages (I–IV) of PPRV. This assay provides a solution with an easy, accurate, rapid and cost-effective PPR diagnostic and partial genome sequencing for use in resource-limited settings.

## 1. Introduction

Peste des petits ruminants (PPR) is a highly contagious viral disease of small ruminants and also clinically affects camels and pigs [1,2,3]. It also affects a large number of wildlife within the order *Artiodactyla,* including critically endangered species [4] and causes sub-clinical infection in cattle and buffaloes [5]. Currently, PPR is endemic in large parts of Africa, the Middle East and Asia, resulting in a significant economic impact for communities relying on these animals as a source of income [1,6,7,8,9]. PPRV was first reported in West Africa in 1942, before it spread to Central and East Africa, Asia and the Middle East [1,7]. It is still spreading globally, with its emergence notably reported in Burundi, China, Georgia, Mongolia, and most recently within the European Union in Bulgaria [10,11,12].

The causative agent, PPR virus (PPRV), is a single stranded negative-sense RNA virus belonging to the *morbillivirus* genus, which includes measles, canine distemper and the recently eradicated rinderpest virus. The PPRV genome is approximately 16 kb long and contains six genes (N-P-M-F-H-L) coding for their respective structural proteins. In addition, two non-structural proteins (C and V) are expressed through leaky scanning and gene editing mechanisms [13]. Four lineages (I–IV) of PPRV based on a sequence comparison of the N (Nucleocapsid) gene have been described [14]. The lineages were initially found in specific geographical areas, lineages I and II were mainly found across Africa, lineage III was found in the Middle East and East Africa whereas lineage IV was only present in Asia, particularly in India. However, lineages are now distributed between the affected regions, with reports of the circulation of more than one lineage in some African countries, specifically in East Africa and Nigeria [9,15,16,17,18,19,20]. PPR has been identified as a target for global control and eradication by 2030 [8]. In order to achieve this goal, a coordinated approach that includes regular vaccination, vigilant surveillance, improved diagnostics and epidemiology, local or regional diagnostic capacity building, coordinated field veterinary services, improved bio-security measures and the control of cross-border animal movement are essential.

PPRV outbreaks in endemic countries are reported throughout the year and usually, the laboratory confirmation of clinical suspicion cases is made by the detection of the PPRV genome in clinical materials using rapid, sensitive and accurate molecular diagnostic techniques such as a reverse transcription-polymerase chain reaction (RT-PCR) that is based on the amplification of parts of the nucleocapsid (N), phosphoprotein (P) or the fusion (F) protein gene [21,22]. In addition, a few real-time RT-PCR (RT-qPCR) techniques targeting the nucleocapsid or matrix (M) protein gene [23,24,25] and loop-mediated isothermal amplification (LAMP) techniques [26,27,28], that require expensive equipment and trained personnel, are also available. The transportation of clinical materials under appropriate conditions from the field to the testing laboratory plays a pivotal role in the outcome of test results as sometimes the viral antigen/RNA deteriorates during the transportation process, making a diagnosis and sequencing difficult. Most of the national diagnostic laboratories use conventional RT-PCR using the N-gene specific primer set NP3/NP4 [22] as the primary method for PPR diagnosis. However, this primer set was designed using the sequence of vaccine strain Nigeria/75/1 which may not be suitable for use in the current scenarios, as there is now more than one lineage of the virus circulating in one country or region, and the issue becomes more difficult to study when a low level of viral infection persists or in the occurrence of the deterioration of viral material during the sample transportation to laboratories or during their storage [19]. Although the RT-qPCR/LAMP methods are more accurate, rapid and sensitive, the end products are not usually suitable for sequencing which is essential for the genetic characterisation of the outbreak virus so as to ascertain the epidemiological linkage between the countries. Therefore, the generation of a standard RT-PCR product with high-quality DNA is necessary for conducting a sequence analysis. Depending on the signal of the amplified DNA on the gel, the PCR products are usually graded as strongly or weakly positive. Generally, this does not affect the confirmation of the clinical suspicion, however, the weakly positive PCR products, which are very common in the case of low-quality/putrified/degraded clinical material, usually fail to produce clean sequence data, resulting in difficulties in the identification of the lineage of the outbreak virus. Therefore, a robust RT-PCR test, with the provision of a nested PCR that can produce high-quality DNA is the ideal starting material for sequencing and, additionally, may help to conduct the successful genetic characterisation of the outbreak viruses. In this paper, we report a direct comparison of the performance of a RT-PCR assay using a new primer set with that of the widely used NP3/NP4 primer pair and, also on the development and validation of a nested PCR to produce high quality DNA from degraded/low-quality RNA for an improved PPR diagnosis and genetic analysis.

## 2. Methods

### 2.1. Viruses and Cells

Seven PPR viruses, representing the four lineages (I–IV), were selected for this study (Table 1). The Vero DogSLAMtag (VDS) cells were infected with the viruses at an multiplicity of infection (MOI) of 0.01, following standard protocol, and were then incubated at 37 °C/5% CO_2_ until an extensive cytopathic effect (CPE) was observed. The virus was harvested following a single cycle of freezing and thawing and stored as single use aliquots at −70 °C. 

### 2.2. Primer Design

In order to design the primers, 114 PPRV full genome sequences (Supplementary Table S1) representing all four lineages were obtained from GenBank (https://www.ncbi.nlm.nih.gov/genbank) accessed on 20 February 2021. Sequences were aligned using the CLUSTALX multiple sequence alignment program [29] from which a highly conserved region was selected in the coding region of the N-gene (nucleotides 1190–1660: KC594074). The primer set NP3/NP4 of Couacy-Hymann and colleagues [22], which is used in diagnostic laboratories worldwide, are also located within this region (nucleotides 1232–1560). In our laboratory, this primer set reasonably detected all four lineages of PPRV with good quality samples, such as the cell culture grown virus or biological samples generated from animal experiments. However, it was difficult to amplify the DNA and sequencing from field samples that were transported across a long distance or that possessed a low level of infection, recorded during the clinical survey. Therefore, our strategy was to design a set of primers outside of the location of the NP3/NP4 primers so that the NP3/NP4 could be used as the nested primer set. The PCR primers were designed from a consensus sequence of 114 sequences (Supplementary Table S1) using the primer BLAST program in NCBI web (https://www.ncbi.nlm.nih.gov/tools/primer-blast accessed on 20 February 2021). A total of three sets of primers (data not shown) were designed to target the highly conserved region (nucleotide position 1190 to 1660) within the N-protein gene and, following the initial screening, one set of primers, N1197F (5′-CTCGGACAGGAGATGGTCAGAAG-3′) and N1658R (5′-CGCGAYCTGAYTGTTGTCTTCTCCC-3′) were taken forward for further testing. The NP3/NP4 primer pair of Couacy-Hymann and colleagues [22] was used as the nested primer in subsequent tests.

### 2.3. RNA Extraction and Real-Time Reverse-Transcription-Polymerase Chain Reaction (RT-qPCR) 

The total RNA was extracted from cell culture grown viruses, infected animal tissues and ethylenediaminetetraacetic acid (EDTA) blood using a TRIzol reagent (Invitrogen, Carlsbad, CA, USA) as previously described [30,31]. In addition, the total RNA from milk samples [32], ocular and nasal swabs collected from sheep and goats from the field [17,18], nasal swabs and faecal samples from experimentally infected animals [33] and environmental samples from a goat market in Nepal [34] were also extracted. For the preparation of a serial dilution of viruses from each lineage, the cell culture grown viruses were diluted 10-fold in a growth media up to 10^−11^ and the total RNA was extracted from each dilution. The RNA was eluted in a final volume of either 40 or 90 uL and stored at −70 °C as single use aliquots until used. RT-qPCR was performed as described previously [25] using a commercial kit (Superscript III Platinum R one step qRT-PCR system kit, Invitrogen, Carlsbad, CA, USA) on an Applied Biosystems 7500 Fast real-time PCR instrument (Applied Biosystems, Thermo Fisher Scientific, Waltham, MA, USA). All the samples were run in duplicate and the samples that presented as positive in only one well were repeat tested for confirmation.

### 2.4. Reverse Transcription (RT), Polymerase Chain Reaction (PCR), Nucleotide (nt) Sequencing

The RT, PCR, sequencing, sequence analysis and assembling were performed as described previously [30] with slight modifications. To summarise, the viral RNA was reverse transcribed using the superscript III first strand synthesis kit (Invitrogen, Carlsbad, CA, USA), according to the manufacturer’s instructions. Eight micro litres of total RNA was used in a total volume of 20 uL RT reaction to maximise detection. In order to compare the performance of both the primer sets for diagnostic purposes, the RNA extracted from 10-fold diluted virus suspensions was used in the RT reaction and the respective cDNA was subsequently used for establishing the PCR reaction. The first round of PCR was performed using primer pairs N1197F/N1658R or NP3/NP4. The weakly positive PCR amplicons obtained from the first PCR using primer pairs N1197F/N1658R were used as the template for the nested PCR using primer pairs NP3/NP4. A KOD hot-start DNA polymerase kit (Novagen, Merck KGaA, Darmstadt, Germany) was used to set up the PCR reaction in a total volume of 25 (first PCR) or 50 uL (nested PCR). A 50 uL reaction consisted of 5 uL of 10× PCR buffer and 2 mM dNTP mixture, 4 uL of 25 mM MgSO_4_, 1 uL each of both a 10 uM forward and reverse primer, 1 uL of polymerase enzyme, 4 uL of the template cDNA/PCR product and the remaining volume was composed of nuclease-free water. Half of the volume of each component was used for a 25 uL reaction that was set up. The PCR conditions were as follows: 2 min at 95 °C; followed by 35 cycles of 20 s at 95 °C (denaturation), 10 s at 55 °C (annealing), and 15 s at 72 °C (extension) with a final extension of 2 min at 72 °C. This cycling condition was used for both the primer sets and for both of the rounds of PCR. Five uL of the PCR products were run on a 1% tris-borate-EDTA (TBE) gel along with a 1 kb plus marker DNA (Invitrogen, Carlsbad, CA, USA) at 100 volt for 30 min for visualization and were subsequently imaged. All the tests were repeated at least twice. For sequencing purposes, the nested PCR amplicons were gel purified when non-specific DNA bands were observed or purified using the QIAEXII PCR purification kit (Qiagen, Venlo, Netherlands) according to the manufacturer’s instructions. Sequencing was conducted using the BigDye^®^ Terminator v3.1 Cycle Sequencing Kit (Applied Biosystems, Carlsbad, CA, USA) using the nested PCR primers (NP3/NP4). Sequences (from the ABI 3730 machine) were assembled and analysed using SeqMan II (DNAStar Lasergene 8.0, DNASTAR, Inc., Madison, WI, USA). Nucleotide sequences of the viruses were aligned using the CLUSTALX multiple sequence alignment program. 

### 2.5. RNA Standards and Dilution Series Used to Determine the Limit of Detection by RT-PCR

The RNA standard for determining the limit of detection by the nested PCR assay was prepared as previously described [33]. Ten-fold serial dilution series (10^9^–10^0^) of the in-vitro transcribed PPRV RNA standards were prepared in nuclease-free water containing a carrier RNA (1 µg mL^−1^) and were tested in RT-PCR using both of the primer sets (N1197F/N1658R and NP3/NP4), followed by a nested PCR using the primer pair NP3/NP4. In addition, ten-fold dilution series (neat virus to 10^−11^) of the RNA, extracted from PPRV strains representing four different lineages (I–IV), were also used to determine the analytical sensitivity of the assay. 

The diagnostic sensitivity was determined using RNA extracted from a total of 26 clinical samples, representing samples from five countries and seven cell-culture grown PPRVs (Table 1). Their diagnostic specificity was assessed using RNA extracted from three different viruses, two morbilliviruses, measles virus and dolphin morbillivirus (MV and DMV), and a pneumovirus, bovine respiratory syncytial virus (bRSV) that infects mainly cattle but can also infect sheep and goats.

## 3. Results and Discussion

PPR, an acute contagious viral disease of wild and domestic small ruminants has been identified for global control and eradication by 2030. Because of its high rate of spread and its expansion beyond its known geographical boundaries, PPR causes significant annual economic losses of between US$1.4 and US$2.1 billion, with around one third of the losses incurred by Africa and a quarter by South Asia [8]. In addition, the losses associated with an outbreak of critically endangered species could be devastating, for example, the outbreak in saiga antelope (*Saiga tatarica mongolica*) in Mongolia [4]. In order to achieve the aim of eradication, a worldwide intensive vaccination program needs to be undertaken to achieve and maintain the required herd immunity levels of >80% in sheep and goats [35,36,37]. Similar to rinderpest, there are two live-attenuated vaccines available for the control of PPR that provide life-long immunity. The Sungri/96 vaccine is mainly used in India whereas the Nigeria/75/1 vaccine is used in the rest of the world. Although progress has been made in controlling the disease, the virus is often reported in new territories, keeping in mind that the majority of the outbreaks in the developing world remain unreported and/are not investigated. Moreover, most of the PPR endemic countries possess less equipped diagnostic laboratories for confirmation of the disease suspicion. Advanced and highly sensitive diagnostic molecular techniques such as RT-qPCR, which acts as the gold standard test for PPR diagnosis requires the provision of expensive equipment and trained personnel to conduct the test and/or analyse the data. Although an appropriate facility to conduct RT-qPCR or LAMP may not be available in every veterinary diagnostic laboratory, a facility to conduct conventional RT-PCR is available in almost all national laboratories, especially in Africa and Asia. 

It would be more efficient to identify the pockets of endemicity responsible for PPRV persistence and to instill higher levels of vaccination immunity in these defined population as was performed in the last phase of rinderpest eradication. Identifying the pockets of endemicity that are responsible for PPRV persistence requires the active surveillance of susceptible animal populations with a highly accurate, rapid and cost-effective diagnostic tool, such as an RT-PCR test. 

### 3.1. Initial Testing 

**Figure 1:**
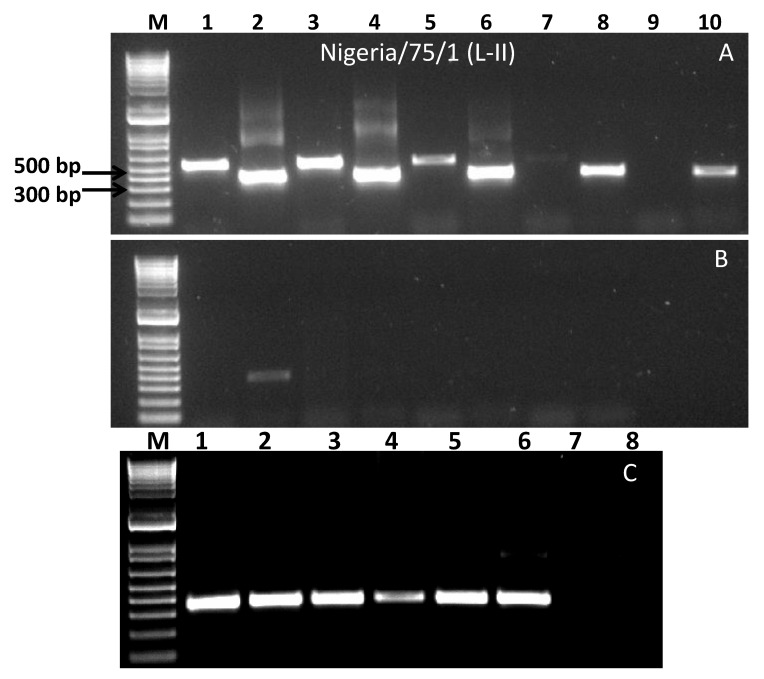
Gel images of PCR products using RNA of Nigeria/75/1 vaccine strain. (**A**,**B**): PCR products obtained from first round of PCR using primer pair NP3/NP4 (even number lane) and N1197F/N1658R (odd number lane), (**C**): nested PCR products using primer pair NP3/NP4. M: 1 kb plus marker DNA; lanes 1–8: PCR products corresponding to dilution 10^−3^ to 10^−10^, respectively. All the odd and even lanes in (**A**,**B**) shows products of primer pair N1197F/N1658R and NP3/NP4, respectively using neat, 10^−1^–10^−10^ RNA. Lanes 1–8 in **C** represents 10^−3^–10^−10^ RNA, respectively.

The primer pair N1197F/N1658R was designed on the conserved region of the C-terminus of the N-gene in such a way that it encompasses the widely used diagnostic primers NP3/NP4. This primer pair generates a product of ~460 bp while NP3/NP4 produces a product of ~350 bp. The primer pairs were initially tested using RNA extracted from the tissue-culture grown lineage II vaccine virus, Nigeria/75/1. As expected, both of the primer sets produced the right sized PCR products, with the NP3/NP4 primer pair showing much higher performance compared to the N1197F/N1658R primer set (Figure 1A,B). The NP3/NP4 primer pair produced right-sized products up to a dilution series of 10^−5^ whereas the N1197F/N1658R primer set generated right-sized products only up to dilution series 10^−2^. This is not surprising as NP3/NP4 primer pair was originally designed on the sequence of the Nigeria/75/1 vaccine strain [22]. However, in the nested PCR, right-sized products were observed for up to dilution series 10^−8^ (Figure 1C). Upon sequencing, clean sequences were obtained from all the nested PCR products. With the above observed success of the use of a new primer pair, further samples of either tissue-culture grown viruses representing the four lineages and field/experimental samples were tested.

### 3.2. Lineage I

**Figure 2:**
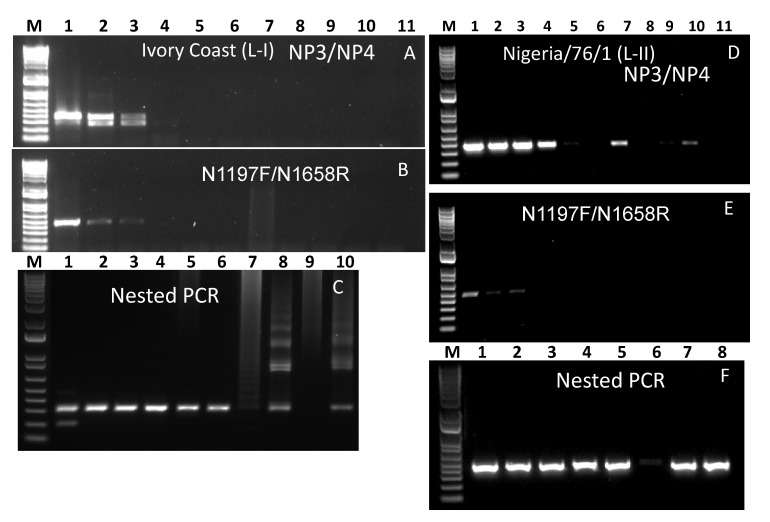
Gel images of PCR products using RNA of lineage I virus Ivory Coast (**A**–**C**) and lineage II virus Nigeria/76/1 (**D**–**F**). (**A**,**D**): PCR products obtained from first round of PCR using primer pair NP3/NP4, (**B**,**E**): PCR products obtained from first round of PCR using primer pair N1197F/N1658R, (**C**,**F**): PCR products obtained from nested PCR using primer pair NP3/NP4. M: 1 kb plus marker DNA; lanes 1–11 in (**A**,**B**,**D**,**E**) correspond to neat, 10^−1^ to 10^−10^ RNA, respectively. Lanes 1–10 in **C** correspond to 10^−1^ to 10^−10^ RNA, and lanes 1–8 in (**F**) correspond to 10^−1^ to 10^−8^ RNA, respectively.

The total RNA extracted from the tissue-culture grown lineage I field virus Côte d’Ivoire was used in this study. PCR products of the right size were observed in cases where the primer set NP3/NP4 had a dilution series of up to 10^−2^, whereas it was up to 10^−1^ for primer pair N1197F/N1658R, although only a very faint band was observed for the dilution 10^−2^ (Figure 2A,B). In the nested PCR, the right sized products were observed up to a dilution series of 10^−7^ after which products of the right size were observed intermittently (Figure 2C). Successful sequencing results were obtained from nested PCR products up to dilution series of 10^−7^.

### 3.3. Lineage II

The total RNA extracted from the tissue-culture grown lineage II vaccine virus (Nigeria/75/1) and the field virus (Nigeria/76/1) were used in this study. The results of the lineage II vaccine virus, Nigeria/75/1, are shown in Figure 1A,B in Section 3.1. Similar to the results of the lineage II vaccine virus, the NP3/NP4 primer pair functioned much better in the field virus (Nigeria/76/1) compared to the N1197F/N1658R primer set. PCR products of the right size were observed in cases where the primer set NP3/NP4 had a dilution series of up to 10^−3^, while it was up to 10^−1^ for primer pair N1197F/N1658R, although only a very faint band was observed for the dilution 10^−2^ (Figure 2D,E). In the nested PCR, right sized products were observed for a dilution series of up to 10^−8^ (Figure 2F). Successful sequencing results were generated from the PCR products up to dilution series 10^−8^.

### 3.4. Lineage III

**Figure 3:**
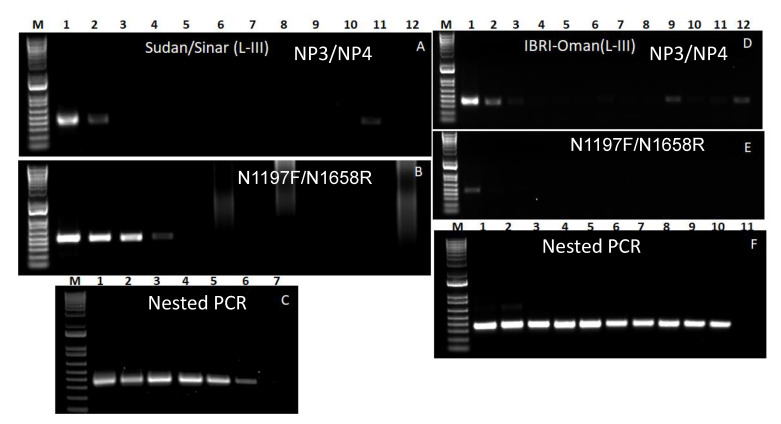
Gel images of PCR products using RNA of Linage III viruses Sudan/Sinar 1972 (**A**–**C**) and IBRI-Oman 1982 (**D**–**F**). (**A**,**D**): PCR products obtained from first round of PCR using primer pair NP3/NP4, (**B**,**E**): PCR products obtained from first round of PCR using primer pair N1197F/N1658R, (**C**,**F**): PCR products obtained from nested PCR using primer pair NP3/NP4. M: 1 kb plus marker DNA; lanes 1–12 in (**A**,**B**,**D**,**E**) correspond to neat, 10^−1^ to 10^−11^ RNA, respectively. Lanes 1–7 in (**C**) correspond to 10^−3^ to 10^−9^ RNA, and lanes 1–11 in (**F**) correspond to neat, 10^−1^ to 10^−10^ RNA, respectively.

The total RNA extracted from two tissue-culture grown lineage III field viruses, Sudan/Sinar/72 and IBRI-Oman/82 were used in this study. Contrary to the results in the case of the lineage II viruses, the N1197F/N1658R primer pair worked much better for the Sudan/1972 virus as compared to the NP3/NP4 primer set. In the case of the Sudan/Sinar/72 virus, PCR products of the right size were observed in cases of the primer set NP3/NP4 had a dilution series of up to 10^−1^ although a very faint band was observed for the dilution 10^−10^ while it was up to 10^−3^ in case of primer pair N1197F/N1658R (Figure 3A,B). The nested PCR result showed amplicons up to the dilution series 10^−8^ (Figure 3C). In the case of the IBRI-Oman/82 virus, PCR products of right size were observed in case of the primer set NP3/NP4 up to a dilution series of 10^−1^ with very faint bands at the dilution series 10^−8^ and 10^−11^, while it was only for neat RNA in the case of the primer pair N1197F/N1658R, albeit with a faint band (Figure 3D,E). In the nested PCR, right-sized products were observed for up to dilution series 10^−9^ (Figure 3F). Successful sequencing results were obtained from the PCR products for up to a dilution series of 10^−7^ in the case of the Sudan/Sinar/72 virus and 10^−9^ in the case of IBRI-Oman/82 virus, respectively.

### 3.5. Lineage IV

**Figure 4:**
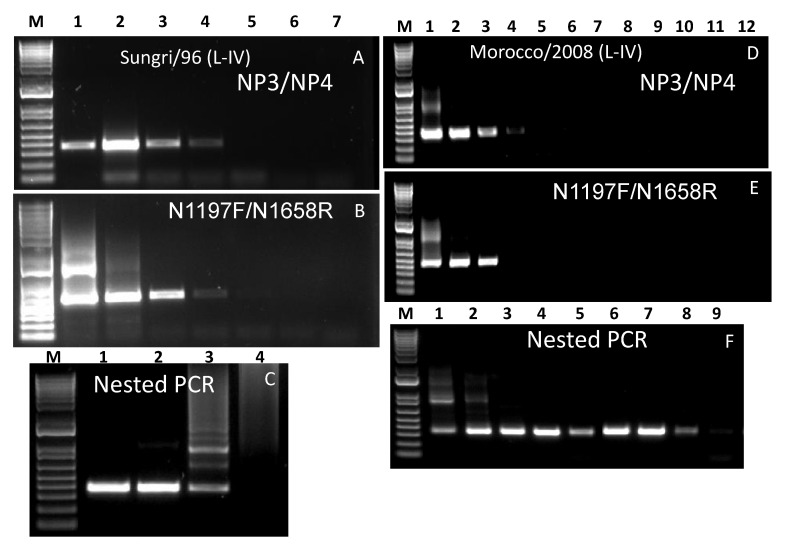
Gel images of PCR products using RNA of lineage IV viruses Sungri/96 (**A**–**C**) and Morocco/2008 (**D**–**F**). (**A**,**D**): PCR products obtained from first round of PCR using primer pair NP3/NP4, (**B**,**E**): PCR products obtained from first round of PCR using primer pair N1197F/N1658R, (**C**,**F**): PCR products obtained from nested PCR using primer pair NP3/NP4. M: 1 kb plus marker DNA; lanes 1–7 in (**A**,**B**) correspond to neat, 10^−1^–10^−7^ RNA, respectively. Lanes 1–4 in (**C**) correspond to 10^−3^–10^−6^ RNA, respectively. Similarly, lanes 1–12 in (**D**,**E**) correspond to neat, 10^−1^–10^−11^ RNA, respectively. Lanes 1–9 in (**F**) correspond to 10^−3^–10^−11^ RNA, respectively.

The total RNA extracted from the tissue-culture grown lineage IV vaccine virus, Sungri/96 and field virus Morocco/2008 were used in this study. In case of the vaccine virus, Sungri/96 PCR products of the right size were observed in the case of both the primer sets, up to a dilution series of 10^−3^, although a relatively faint band was observed on the dilution 10^−3^ (Figure 4A,B). For the field virus Morocco/2008, the performance for both the primer sets were also similar, although a very faint band was observed in dilution series 10^−3^ for primer set NP3/NP4 (Figure 4D,E). In the nested PCR, the right sized products were observed for a dilution series of up to 10^−5^ and 10^−11^ in the case of the Sungri/96 and Morocco/2008 virus, respectively (Figure 4C,F). Successful sequencing results were obtained from the PCR products for up to dilution series 10^−5^ and 10^−10^ in case of the Sungri/96 and Morocco 2008 virus, respectively.

### 3.6. RNA Standards and Dilution Series Used to Determine Analytical Sensitivity of the Nested PCR Assay

**Figure 5:**
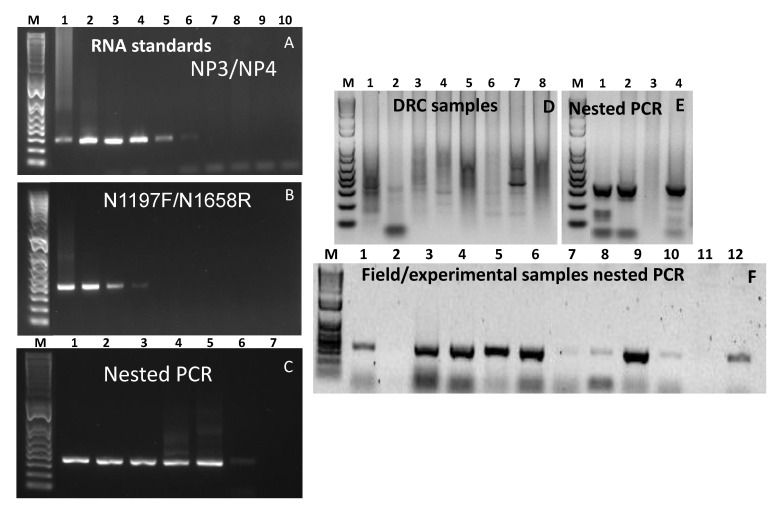
Gel images of PCR products using in-vitro transcribed RNA standards (**A**–**C**) and field/experimental samples (**D**–**F**). (**A**): PCR products obtained from first round of PCR using primer pair NP3/NP4, (**B**): PCR products obtained from first round of PCR using primer pair N1197F/N1658R, (**C**): PCR products obtained from nested PCR using primer pair NP3/NP4, (**D**): PCR products obtained from first round of PCR using primer pair NP3/NP4 (lanes 1–4) and N1197F/N1658R (lanes 5–8) of samples from Democratic Republic of Congo (DRC) (correspond to samples 1–4 in Table 1), (**E**): nested PCR products of DRC samples using primer pair NP3/NP4, (**F**): nested PCR products of field or experimental samples (correspond to samples 5–16 in Table 1) using primer pair NP3/NP4. M: 1 kb plus marker DNA; lanes 1–10 in (**A**,**B**) correspond to 10^9^–10^0^ genome copies, respectively. Lanes 1–7 in (**C**) correspond to 10^6^–10^0^ genome copies, respectively.

Using in vitro synthesized RNA standards, the limit of the detection of the RT-PCR assay using primer pair NP3/NP4 was found to be much higher, of up to 10^4^ copies, while that of primer pair N1197F/N1658R was 100-fold lower (only up to 10^6^ copies) although the products on the last dilution for each primer set were relatively faint (Figure 5A,B). However, when nested PCR was conducted, right sized products were obtained for up to 10^1^ RNA copies (Figure 5C), although, once again, the product on the 10^1^ copies was very faint and did not produce a clean sequence result. This indicates that using high quality RNA as the starting material for 10 copies of the PPRV genome was sufficient for a diagnosis, while a minimum of 100 copies of the genome are required for obtaining clean sequence results. However, this could be much higher in cases where field samples are used, as the RNA quality is also critical. The transportation of clinical materials under appropriate conditions from the field to the testing laboratory plays a pivotal role in the outcome of test results, as sometimes the viral antigen/RNA deteriorates during the transportation process, making diagnosis difficult.

### 3.7. Testing Clinical Samples from the Field and Animal Experiments

**Figure 6:**
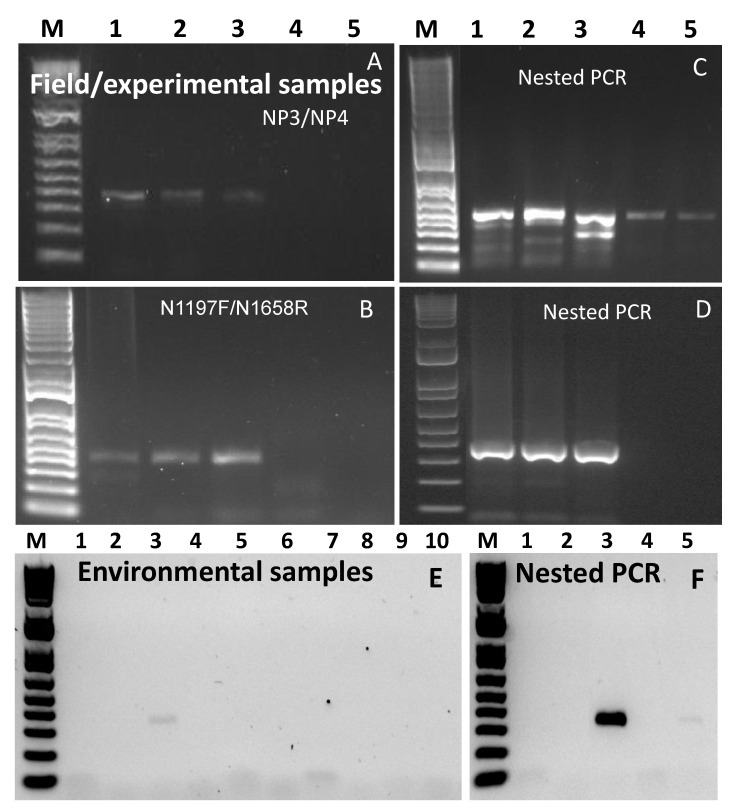
Gel images of PCR products using field/experimental (correspond to samples 17–21 in Table 1) samples (**A**–**D**) and environmental (correspond to samples 22–26 in Table 1) samples (**E**,**F**). (**A**): PCR products obtained from first round of PCR using primer pair NP3/NP4, (**B**): PCR products obtained from first round of PCR using primer pair N1197F/N1658R, (**C**): nested PCR products using primer pair NP3/NP4, (**D**): nested PCR products using primer pair NP3/NP4 when template DNA was purified, (**E**): PCR products obtained from first round of PCR using primer pair NP3/NP4 (lanes 1–5) and N1197F/N1658R (lanes 6–10) of environmental samples from Nepal (correspond to samples 22–26 in Table 1), (**F**): nested PCR products of environmental samples using primer pair NP3/NP4. M: 1 kb plus marker DNA.

A total of 21 clinical samples consisting of nasal/ocular swabs, blood, milk, faeces and tissues were used in this study. Of the four nasal swabs collected from DRC, the first PCR, using any of the primer pairs, did not generate any PCR products of right size (Figure 5D). However, when nested PCR was carried out PCR products of the right size were observed in three of them, albeit with some non-specific products (Figure 5E). Similar results were obtained when a further 12 samples, corresponding to samples 5–16 in Table 1, were tested (Figure 5F). Nucleotide sequencing was successful in all the amplicons, exhibiting a strong signal, however, the products with non-specific bands required gel purification. Similar results were observed when PCR products using field/experimental corresponding to samples 17–21 in Table 1 were tested, some samples produced the right sized PCR products in the first PCR (Figure 6A,B) which was perfect for diagnosis, however failed to do so when subjected to sequencing. This could be due to the poor quality of the DNA generated in the first PCR as they were weakly positive. When nested PCR was carried out, stronger bands were observed albeit with many non-specific products, including a band of ~450 bp (Figure 6C) indicating the primer pair N1197F/N1658R to still be functional in the nested PCR. The PCR product from first PCR-using primer pair N1197F/N1658R were purified, and the purified PCR product was subsequently used as the template in the nested PCR. This produced a clean PCR product of the right size from which nucleotide sequence was generated successfully. It is noteworthy that the sequence obtained from the gel purified PCR products from Figure 6C,D were identical. All the tests were repeated at least twice and produced similar results.

### 3.8. Testing Environmental Samples

In a previous study, we used the diagnostic RT-PCR using an NP3/NP4 primer pair, to detect the PPRV genome in environmental samples [34]. Samples with very low C_T_-values produced products that showed right-sized, albeit weak, DNA bands on the gel, indicating that the detection of a PPRV genome in these samples was feasible, however, it failed when subjected to sequencing, possibly due to the poor quality of the DNA [31]. Therefore, five environmental samples that were collected from a goat market with a range of C_T_-values (24.61–31.29) were tested in this study to evaluate the nested PCR to generate sequence data. Of the five samples tested, only one sample with the lowest C_T_-value produced a very weak band in the first PCR, using primer pair NP3/NP4 while no band was produced when primer pair N1197F/N1658R was used (Figure 6E). However, the nested PCR generated a strong DNA band on which sequencing was successful. Moreover, a second sample became positive on the nested PCR (Figure 6F, lane 5), thereby enhancing the diagnostic sensitivity. All the tests were repeated at least twice, and produced similar results.

Thus, the newly developed nested PCR enhances the diagnostic sensitivity and facilitates the determination of the lineages of the circulating virus, particularly in low viral load samples.

### 3.9. Specificity Test 

Diagnostic specificity was assessed using the RNA extracted from three different viruses, two morbilliviruses (MV and DMV), and one pneumovirus, bRSV that causes respiratory disease mainly in cattle but is also capable of infecting sheep and goats. The PPRV-specific primer sets demonstrated a high degree of specificity for PPRV. All seven PPRV isolates were amplified by this assay, whereas the RNA extracted from the three viruses (two closely related morbilliviruses, MV and DMV; and one pneumovirus, (bRSV)) yielded negative results (Table 1).

## 4. Conclusions

In conclusion, the study demonstrates that RT-PCR, using the new primer set N1197F/N1658R at the C-terminus of the N-gene for initial amplification, and then using widely used diagnostic primers NP3/NP4 as the primer set for the nested PCR, can improve the diagnostic capacity and allows for the conduction of a genetic analysis on weakly positive/degraded samples, and even on environmental samples collected from animal markets where a low level of viral RNA is present. This nested PCR will enhance the capacity building of national and regional laboratories in the developing world, especially in resource-limited settings to diagnose PPR in a timely manner, and, accordingly, immediate control measures could be implemented to prevent the further spread of the virus to PPR-free regions within the same country and, also the transboundary movement of the virus which is critical for the ongoing eradication programme.

## Figures and Tables

**Table 1:** Virus isolates and clinical samples used for the development and evaluation of the nested PCR. Samples 1–21 were clinical samples either collected from the field (Algeria, Bangladesh, Democratic Republic of Congo (DRC), Israel and Tanzania) or experimental samples. The samples with an asterisk (*) at the end of their name (10–11, 16, 19 and 21) were collected from goats experimentally infected with PPRV (Morocco/2008) at The Pirbright Institute (TPI). Samples 22–26 are environmental samples collected from a goat market in Nepal. Samples 27–33 are cell-culture grown PPR viruses comprising four lineages. The lineages of the PPR viruses are shown in parenthesis under sample details wherever applicable. The lineages of the field samples identified by sequencing the nested PCR products in this study are underlined. ^#^ indicates mean C_T_-values obtained from real-time RT-PCR (RT-qPCR) assay. -: negative; + and ++: weakly and moderately positive, respectively, but nucleotide (nt) sequencing was not possible from these products; +++: strongly positive and nt sequence was generated from these products.

Serial No.	Sample Details	Mean C_T_^#^	NP3/NP4	N1197F/N1658R	Nested PCR
1	DRC nasal swab-goat-5 (L-III)	24.79	-	-	+++
2	DRC nasal swab-sheep-10 (L-III)	25.09	-	-	+++
3	DRC nasal swab-goat-3	32.79	-	-	-
4	DRC nasal swab-goat- 13 (L-III)	22.17	-	-	+++
5	Tanzania occular swab-goat-16	26.37	-	+	++
6	Tanzania occular swab-goat-11	36.96	-	-	-
7	Tanzania occular swab-goat-10 (L-II)	22.96	+	+	+++
8	Algeria Farm 1-blood 2 (L-IV)	22.05	+	+	+++
9	Israel Field lung tissue-1 (L-IV)	20.62	+	+	+++
10	Faecal sample-5 * (L-IV)	22.45	+	+	+++
11	Nasal swab-2 *	32.65	-	-	-
12	Tanzania nasal swab-sheep-5	31.04	-	-	+
13	Bangladesh Milk-B19/Nihkanchari/2015 (L-IV)	23.54	+	+	+++
14	Bangladesh Milk-B53/Savara/2015	30.1	-	-	+
15	Israel Field lung tissue-3	no C_T_	-	-	-
16	Nasal swab-5 *	28.35	-	-	++
17	Algeria Farm 1-blood 1 (L-IV)	25.23	+	+	+++
18	Algeria Farm 1-blood 5 (L-IV)	26.34	+	+	+++
19	Nasal swab-4 * (L-IV)	25.18	+	++	+++
20	Tanzania nasal swab-goat-2	32	-	-	-
21	Nasal swab-3 *	31.22	-	-	-
22	Trb/Nepal/goatmarket/pen1/metalwall	27.55	-	-	-
23	Trb/Nepal/goatmarket/pen2/woodenpost/b	31.29	-	-	-
24	Trb/Nepal/goatmarket/pen1/metalpole/c (L-IV)	24.61	+	-	+++
25	Trb/Nepal/goatmarket/pen2/metalpole/a	29.68	-	-	-
26	Trb/Nepal/goatmarket/pen1/woodenpost/b	26.05	-	-	+
27	PPRV/Ivory coast (L-I)	20.30	+++	+++	+++
28	PPRV/Nigeria/75/1 (L-II)	19.24	+++	+++	+++
29	PPRV/Nigeria/76/1 (L-II)	23.68	+++	+++	+++
30	PPRV/IBRI-Oman/82 (L-III)	19.84	+++	+++	+++
31	PPRV/Sudan/Sinar/72 (L-III)	12.03	+++	+++	+++
32	PPRV/Sungri/96 (L-IV)	13.45	+++	+++	+++
33	PPRV/Morocco/2008 (L-IV)	16.85	+++	+++	+++
34	Dolphin Morbillivirus (DMV)	No C_T_	-	-	-
35	Measles virus (MV)	No C_T_	-	-	-
36	Bovine RSV (bRSV)	No C_T_	-	-	-

## Data Availability

Not applicable.

## Supplementary Materials

The following are available online at https://www.mdpi.com/article/10.3390/ani11113170/s1, Table S1: List of PPRV genome sequences used to design the primers in this study.
